# Initial Staging of Hodgkin’s Disease: Role of Contrast-Enhanced ^18^F FDG PET/CT

**DOI:** 10.1097/MD.0000000000000050

**Published:** 2014-08-04

**Authors:** Agostino Chiaravalloti, Roberta Danieli, Cristiana Ragano Caracciolo, Laura Travascio, Maria Cantonetti, Andrea Gallamini, Manlio Guazzaroni, Antonio Orlacchio, Giovanni Simonetti, Orazio Schillaci

**Affiliations:** Department of Biomedicine and Prevention, University Tor Vergata, Rome (AC, RD, CRC, LT, MC, MG, AO, GS, OS); Azienda Ospedaliera S. Coce e Carle, Hematology, Cuneo (AG); and IRCCS Neuromed, Pozzilli (OS), Italy.

## Abstract

The objective of this study was to compare the diagnostic accuracy of positron emission tomography/low-dose computed tomography (PET/ldCT) versus the same technique implemented by contrast-enhanced computed tomography (ceCT) in staging Hodgkin’s disease (HD).

Forty patients (18 men and 22 women, mean age 30 ± 9.6) with biopsy-proven HD underwent a PET/ldCT study for initial staging including an unenhanced low-dose computed tomography for attenuation correction with positron emission tomography acquisition and a ceCT, performed at the end of the PET/ldCT scan, in the same exam session. A detailed datasheet was generated for illness locations for separate imaging modality comparison and then merged in order to compare the separate imaging method results (PET/ldCT and ceCT) versus merged results positron emission tomography/contrast-enhanced computed tomography (PET/ceCT). The nodal and extranodal lesions detected by each technique were then compared with follow-up data that served as the reference standard.

No significant differences were found at staging between PET/ldCT and PET/ceCT in our series. One hundred and eighty four stations of nodal involvement have been found with no differences in both modalities. Extranodal involvement was identified in 26 sites by PET/ldCT and in 28 by PET/ceCT. We did not find significant differences concerning the stage (Ann Arbor).

Our study shows a good concordance and conjunction between PET/ldCT and ceCT in both nodal and extranodal sites in the initial staging of HD, suggesting that PET/ldCT could suffice in most of these patients.

## INTRODUCTION

Hodgkin’s disease (HD) is a lymphoproliferative disorder presenting an incidence in the United Kingdom and the United States of 2.7–2.8 per 100,000^1^ and the proper staging of the disease, that is the aim of our study, is actually of great impact when planning radiotherapy^2,3^ and chemotherapy (CHT).^4^ In particular, in HD the CHT treatment consisting of adriamycin, bleomycin, vinblastine, dacarbazine (ABVD) greatly differs in toxic collateral effects from that of other CHT schemes as bleomycin, etoposide, adriamycin cyclophosphamide, vincristine (Oncovin), procarbazine, prednisone regimens that is usually reserved in selected case of advanced disease.^5^ Recent studies showed that the principal short-term toxic effect of ABVD treatment is represented by neutropenia and hair loss^6,7^ while skin, liver, and lung complications are reported in a few patients only^6–9^ and no permanent impairment of brain glucose metabolism has been reported in patients treated with this CHT.^10^

The Ann Arbor system is used to stage HD taking into account the sites of involvement and systemic symptoms due to lymphoma.^11^ As far as staging imaging procedure is concerned, 2-deoxy-2-(F18) fluoro-d-glucose positron emission tomography/computed tomography (^18^F FDG PET/CT) provides crucial metabolic information in staging lymphoma, adding functional features to morphologic staging^12^ and modifying the treatment strategy in one-third of HD patients when compared with other imaging modalities.^12^ Contrast-enhanced computed tomography (ceCT, with oral and contrast administration) lack good sensitivity while evaluating lymph nodal disease, and intravenous contrast administration does not present an added value when investigating bone marrow involvement.^13^ Integrated positron emission tomography/contrast-enhanced computed tomography (PET/ceCT), in which a full-ring-detector clinical positron emission tomography (PET) scanner and ceCT are combined, makes it possible to acquire both metabolic and anatomic imaging data using a single device in a single diagnostic session and provides precise anatomic localization of suspicious area of increased 2-deoxy-2-(F18) fluoro-d-glucose (^18^F FDG) uptake. The additional availability of ceCT data increases the diagnostic accuracy of positron emission tomography/computed tomography (PET/CT), especially when clear anatomic information are required (as in the case of head and neck and pelvic cancer).^14–16^ In particular, as compared with positron emission tomography/low-dose computed tomography (PET/ldCT), PET/ceCT shows an elevated diagnostic accuracy for lymph node staging in patients with rectal cancer^15^ and allows a more definitive diagnosis in laryngeal carcinoma.^16^

To the best of our knowledge, few studies have investigated the conjunction of PET/ldCT with ceCT for staging HD. Most of the papers in this field show that PET/ceCT is able to improve the diagnostic accuracy in the assessment of HD in spleen and liver, leading to a significantly more intensive treatment in these patients,^17,18^ whereas in other studies the differences at staging did not reach statistical significance showing a good correlation between the different imaging modalities (PET/ceCT and PET/ldCT).^19,20^

The aim of this study is the evaluation of the differences at staging for both nodal and extranodal sites of HD localization by means of PET/ldCT and PET/ceCT performed in the same examination session. Next, we investigated the performance of PET/ldCT and PET/ceCT compared with ceCT alone because, to the best of our knowledge, this imaging modality is often performed in clinical routine especially for its availability and relatively low costs.

## MATERIALS AND METHODS

### Patients

Forty patients (18 men and 22 women, mean age 30 ± 9.6 years old) with biopsy-proven HD^21^ underwent a PET/ldCT and a ceCT for staging HD in the same examination session. Patients with other oncologic or HIV history were excluded from the study. No patient was suffering from liver or renal disease, nor was any patient pregnant or breastfeeding.

After 20 ± 5 days post first-line CHT (ABVD×2 cycles—dose intensity 100%—that consist of doxorubicin 25 mg/m^2^ iv, Bleomycin 10,000 units/m^2^, Vinblastine 6 mg/m^2^, and dacarbazine 375 mg/m^2^ for 2 mo) all the patients were evaluated in order to assess treatment response (see below).^22^ The study has been approved by the local ethics committee and a written informed consent has been obtained in all cases from the patients themselves in accordance with the Declaration of Helsinki.^23^

### PET/ldCT Scanning

All patients fasted for at least 5 hours before ^18^F FDG intravenous injection; serum glucose level was normal in all of them (≤107 mg/mL). As already reported in our similar study in this field,^10,24^ patients were injected with 370–450 MBq of ^18^F FDG intravenous and hydrated (500 mL of iv saline sodium chloride, 0.9%) to reduce pooling of the radiotracer in the kidneys.

The PET/CT system Discovery ST16 (GE Medical Systems, TN) was used for the whole population under examination.^10,24,25^ The system combines a high-speed ultra 16-detector-row (912 detectors per row) computed tomography (CT) unit and a PET scanner with 10,080 bismuth germanate crystals in 24 rings. Axial FWHM 1 cm radius is 5.2 mm in 3-dimensional (3D) mode and axial field of view (FOV) is 157 mm. For the PET/ldCT a low-amperage CT scan was acquired for attenuation correction of PET images (80 mA, 140 kV, FOV about 420–500 mm, and CT slice thickness 3.75). The computed tomography dose index (CTDI) for low-dose computed tomography (ldCT) was 4.0175 (±0.84) mGy and the dose-length product (DLP) was 473.296 (±161.09) mGy-cm. After nonenhanced CT, total-body PET examination in the caudocranial direction from upper thighs to vertex was performed (3.5 min per bed). Reconstruction was performed using the 3D reconstruction method of ordered subset expectation maximization with 30 subsets and 2 iterations.^24^

### ceCT Scanning

At staging, ceCT scan with 120–140 kV, automatic milliampere (limit 330–350 mA), thickness 3.750 mm reconstructed at 1.25 mm, acquisition mode 27.50/1.375:1, gantry rotation time 0.6 s, large FOV, matrix 512 × 512) was carried out with intravenous administration of nonionic iodinated contrast material (100–120 mL, 370 mgI/mL, 420 mgI/kg at 3 mL/s), obtaining 2 successive stacks of scans. In order to investigate the presence of any rapid/low enhancing lesion of liver or kidneys, the first comprised the upper abdomen with a 30-second delay from the injection onset leading to 18.85 (±0.14) mGy for CTDI and 883.11 (±161.40) mGy-cm for DLP; the second extended from the neck to the pelvis with a 60-second delay leading to 18.5 (±0.47) mGy for CTDI and 1607.88 (±148.02) mGy-cm for DLP. Brain ceCT was also obtained 3 minutes after intravenous contrast administration: CTDI = 80.76 (±0.02) mGy and DLP = 1292.19 (±0.02).^26^

### Image Analysis

**FIGURE 1:**
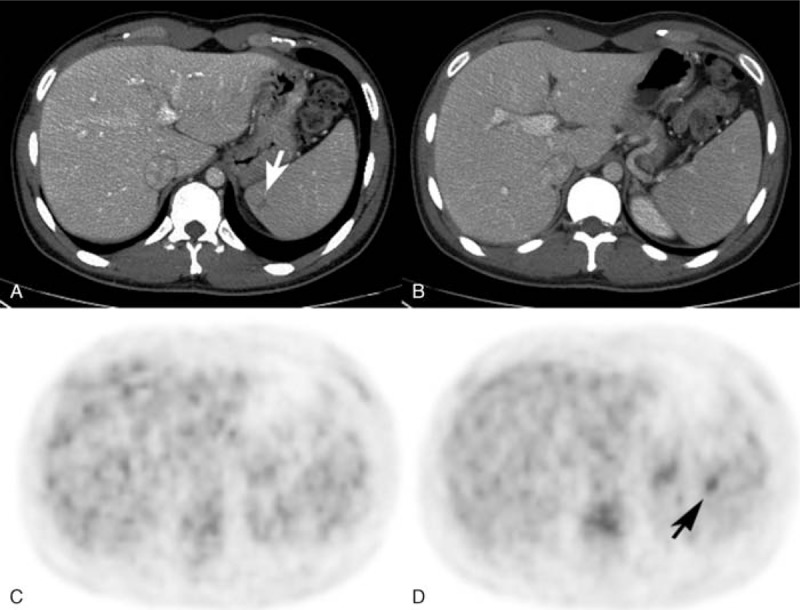
(A) A patient with a hypodense HD lesion in the spleen (arrow) and (B) another patient with no abnormalities in ceCT. (C) No pathological ^18^F FDG uptake was detectable in the patient shown in (A) and (C), while in (D) a focal lesion was detectable in PET/ldCT (arrow) in the patient with a normal ceCT scan in the spleen (B). All these findings were not detectable after 2×ABVD cycles (see text).

**FIGURE 2:**
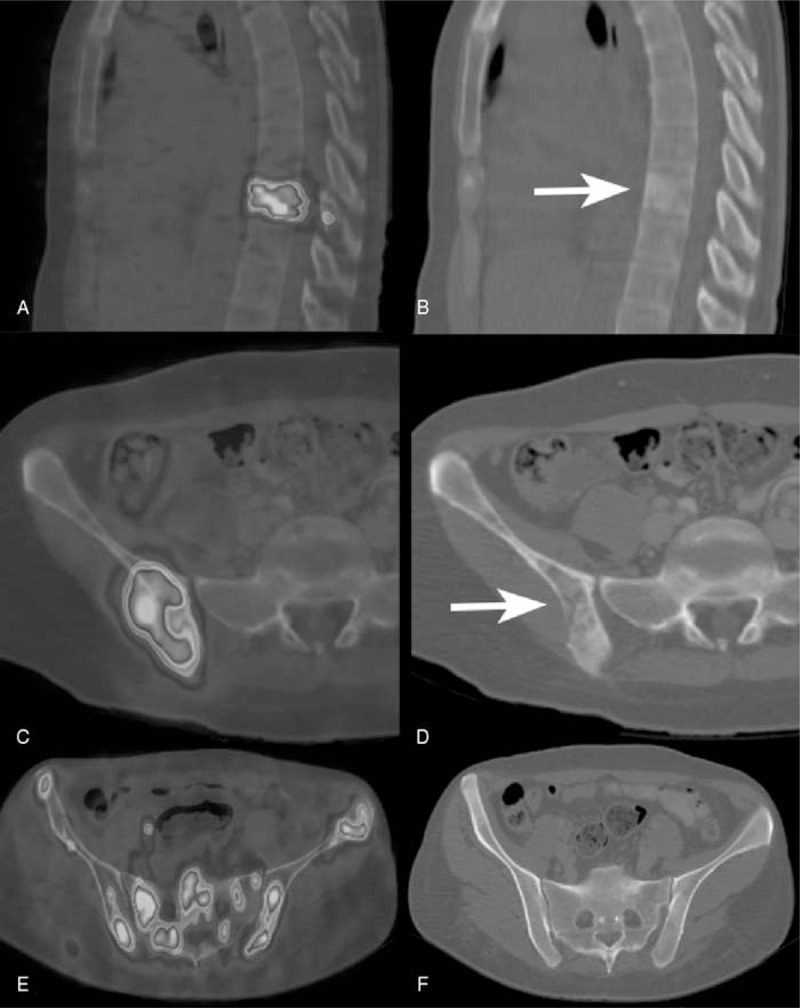
(A) Focal ^18^F FDG uptake in a thoracic vertebrae corresponding to a sclerotic lesion that appears hyperdense and irregular in ceCT (B). Increased ^18^F FDG uptake in the right ilium (C) corresponding to a lytic area (soft-tissue attenuation with irregular margins) in ceCT (D). Diffuse ^18^F FDG uptake in the pelvis (E) in the absence of morphological abnormalities in ceCT (F).

The nuclear medicine physician and the radiologist were unaware of the PET/ldCT and ceCT results, respectively. According to other similar reports in this field,^18^ the visual analysis of PET/ldCT and ceCT images has been performed on a dedicated workstation by a nuclear physician and a radiologist, both aware of the clinical history of the patient. Any focus of increased ^18^F FDG uptake over background not located in areas of normal ^18^F FDG uptake (central nervous system, heart, digestive tract, thyroid, and muscles) and/or excretion (urinary tract) was considered positive for tumor (Figure 1 and Figure 2).

For ceCT, standard CT criteria for individual lymph node groups (when >10 mm in short axis), lung, liver, and spleen were used to determine the site of suspect HD localization.^27^ For bone marrow involvement, any lytic area that usually appears as a region of soft-tissue attenuation with irregular margins that usually breach the cortex or any sclerotic lesions that appear hyperdense and irregular has been considered pathologic (Figure 2).

For nodal involvement, a datasheet indicating the nodal stations was filled for PET/ldCT and ceCT results. As far as the extranodal site is concerned, the physicians were required to sign the presence or absence of disease (regardless of diffuse or focal) in lung, liver, spleen, bone marrow, skin, and brain. A third datasheet was then generated, including functional (PET) and ceCT data. The clinical stage of the patients was assessed in agreement with Ann Arbor classification.^11^

### Follow-up Data as the Reference Standard

Any area of residual ^18^F FDG uptake in interim PET has been evaluated by 2 experienced nuclear medicine physicians (AC and OS) by means of qualitative analysis according to the Deauville 5 point score.^22^ All the positive findings in interim PET have been confirmed by means of mediastinum or upper cervical lymph node(s) biopsy (because these were the sites of no-therapy response or recurrence detected in PET/CT).

All the findings detected in staging PET/ldCT, ceCT, and PET/ceCT were compared with those obtained after CHT: the absence or decrease of ^18^F FDG uptake and the decrease of lesion size and pathologic shape for ceCT data has been used for further confirmation of the pathological findings in the first scan (Table 1).

**TABLE 1:**
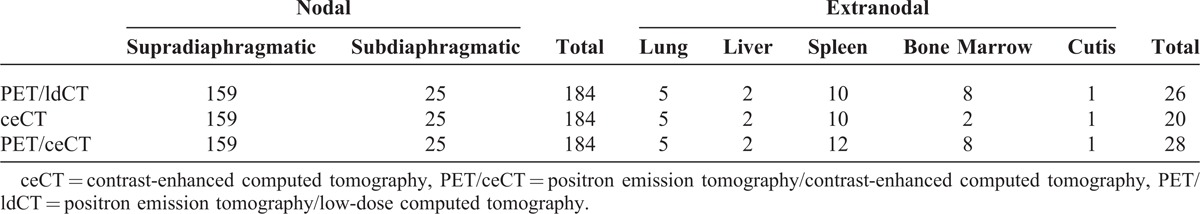
Outline of PET/ldCT, CeCT, and PET/ceCT Findings That Include the Nodal Stations (Divided Into Supra- and Subdiaphragmatic) and Extranodal Involvement (Regardless of Diffuse or Focal Lesions)

### Statistical Analysis

Agreement among techniques has been studied with the κ-statistic. In order to assess the statistical significance on extranodal findings by different imaging methods, we performed *P* calculation by means of Fisher’s exact test. In order to evaluate the impact of different imaging modalities on staging, we performed a two-way analysis of variance test. A hypothesis was considered valid when *P* value was ≤0.05.

## RESULTS

There is a good agreement between PET/ldCT and PET/ceCT (95.14% of the observations, κ = 0.939). By means of PET/ceCT, 212 sites of both nodal and extranodal illness localization were found; 210 were detected by PET/ldCT and 204 by ceCT alone. As far as nodal involvement is concerned (184 lymph nodes, 40 patients), there was complete concordance among the 3 imaging modalities (Table 1). We did not find any difference between ldCT and ceCT in supra- and subdiaphragmatic lymph node sites (regional analysis), lung, skin, and bone marrow involvement, whereas liver and spleen sites were not detectable in ldCT.

PET/ldCT detected 26 extranodal lesions, ceCT alone detected 20 lesions and 28 were detected by PET/ceCT (Table 1, Figure 1). No statistically significant difference has been found comparing PET/ldCT and ceCT in the detection of extranodal involvement (*P* = 0.0776). One patient presented a spleen lesion detectable only with ceCT, whereas another presented a spleen lesion detectable only with PET/ldCT (Figure 1). While comparing PET/ldCT and PET/ceCT results, no differences have been found for extranodal disease involvement (*P* = 1). PET/ceCT detect more extranodal lesions than ceCT alone (28 vs 20 lesions, *P* = 0.0044) as shown in Table 1.

Eight patients (20% of the entire population) presented bone marrow involvement. All these patients presented positive findings in PET/ldCT and PET/ceCT, whereas only 2 of them (5% of the entire population, 25% of the patients with bone marrow involvement) were positive in ceCT (*P* = 0.007) (examples are shown in Figure 2). Both brain PET/ldCT and ceCT were negative for HD, and superior mediastinum was the most frequent localization of HD in our series (34 patients, 85%).

Regarding the staging, 6 patients (15%) were stage I, 15 patients (37.5%) stage II, 3 patients (7.5%) stage III, and 13 patients (40%) stage IV in PET/ceCT (Table 2).

**TABLE 2:**
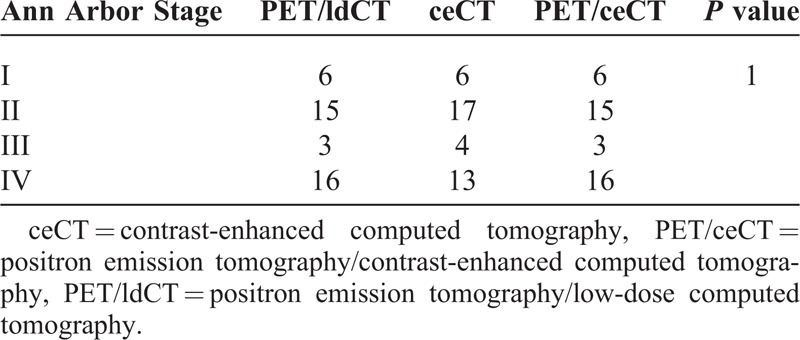
Stage of the Disease Defined by PET/ldCT, CeCT, and PET/ceCT

**FIGURE 3:**
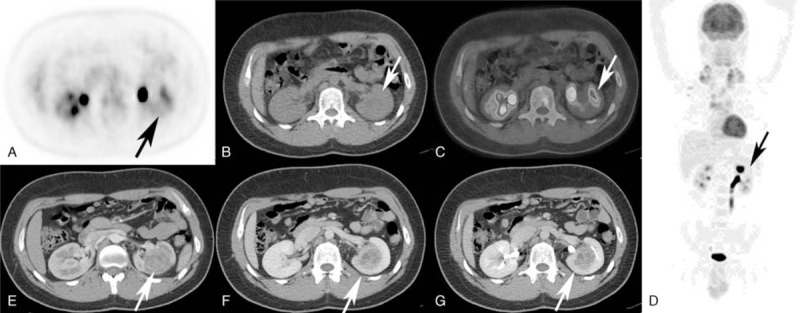
Incidental finding of a CCRCC of the left kidney in a 23-year-old male patient. (A) There are no significant abnormalities of ^18^F FDG distribution in the left kidney (arrow), and (B) no abnormalities are detectable in ldCT and (C) PET/ldCT (arrows). Whole body PET scan of the ^18^F FDG distribution in the patient examined (stage II) (D); the arrow indicates the site of the lesion. ceCT of the upper abdomen with a 30-second (E) and 60-second (F) delay from intravenous contrast media administration shows a lesion with irregular enhancement because of areas of necrosis (arrows). (G) The excretory phase (only for illustrative purposes, see text) performed for the assessment of the collecting system anatomy; the arrow indicates the site of the lesion.

As outlined in Table 2, there were no statistically significant differences between the imaging modalities at staging (F = 0, *P* = 1). Disagreement about the stage of the disease between PET/ldCT and ceCT was found in 3/40 patients (7.5%), which showed bone marrow involvement. According to ceCT results, 2 of these 3 patients were stage II and 1 was stage III, whereas they were stage IV in PET/ldCT and PET/ceCT. As a collateral finding, 1 patient (man, 23 years old) showed a lesion in the left kidney that was consistent with a clear renal cell carcinoma (CCRCC, Figure 3).

## DISCUSSION

One of the main findings of our study is a good concordance at staging between PET/ldCT and PET/ceCT in the detection of nodal and extranodal HD involvement. As outlined in Table 1, the main differences with ceCT are due to bone marrow sites where intravenous contrast administration cover a minor role.^13^ Hence, the lack of differences between PET/ldCT and PET/ceCT is rather a result of PET than CT-imaging protocols. During image evaluation, the radiologist did not report any difference between the number of regions of supra- and subdiaphragmatic lymph node sites in ldCT and ceCT, respectively. However, if one considers the number of lymph node sites, in our experience, ceCT is able to detect a larger number of lymph nodes as compared to ldCT.

An issue recently outlined is the opportunity of using oral and intravenous contrast agents during a PET/CT study, as they may lead to misinterpret PET/CT examinations, while providing better anatomical details and showing contrast-enhancing lesions.^28^ Intravenous contrast agents have been reported to provoke artifacts at PET/CT scans due to the transient bolus passage of undiluted intravenous contrast agent,^29^ and some authors proved that PET/ldCT (without oral or iv contrast agents) is feasible to stage HD and non-Hodgkin lymphoma (NHL) as well.^18^ To date, several PET/ceCT protocols have been proposed.^30^ In particular, Brix et al^30^ investigated a biphasic injection of intravenous contrast (90 and 50 mL at 3 and 1.5 mL/s, respectively) versus a triple-phase injection (90, 40, and 40 mL at 3, 2, and 1.5 mL/s, respectively) in the craniocaudal direction with a 50-second delay and a dual-phase injection (80 and 60 mL at 3 and 1.5 mL/s, respectively) in the caudocranial direction with a 50-second delay. The authors concluded that a dual-phase intravenous contrast injection and a CT in the caudocranial direction with a 50-second delay yields the best high image quality in absence of contrast-related artifacts on CT images with reproducible high levels of PET image quality after CT-based attenuation correction using the ceCT images.^30^ In another report of Pfannenberg et al,^31^ a ceCT consisting of a multiphase CT protocol including a low-dose nonenhanced attenuation scan and an arterial and portal–venous ceCT scan followed by a whole body PET was of additional value in 52/100 patients (85 total lesions) and changed the PET/CT interpretation in 42% of the patients. To note, only 6 patients were affected by lymphoma in this study, whereas most of the patients examined were affected by a large variety of cancer (gastrointestinal, bronchial, neuroendocrine, head-neck cancer, and so on).^31^ In these patients, the incremental benefit of diagnostic CT is due to the correct localization of gastrointestinal and peritoneal lesions (due to the improved delineation of the bowel wall by oral and rectal negative contrast agents in combination with standard CT dose acquisition) or in differentiating malignant FDG uptake from nonmalignant and physiological uptake in infectious lesions, splenosis, postoperative changes, and sites of physiological FDG uptake in the bowel and bladder by the typical CT morphology.^31^

During the execution of a PET/ceCT examination, the patients incur an increased exposure compared with an individual ceCT or PET/ldCT examination.^32^ Our study was not designed to estimate the radiation exposure in the different imaging modalities used; nevertheless, some conclusions can be drawn from the different protocols used in our study. In fact, the PET/ceCT protocol used is similar to 3 of the 4 PET/CT protocols investigated by Brix et al^30^ where separate ldCT scans were acquired for attenuation correction of emission data in addition to a ceCT; this study shows a higher radiation exposure in these patients, mainly due to higher milliampere and kilovolts of ceCT, with an effective dose of 26.4, 24.4, and 23.7 mSv, respectively, that is higher as compared to a PET/ldCT (effective dose 8.5 mSv).^30^

In a previous published study, Picardi et al^17^ compared the role of PET/ceCT and PET/ldCT in 2 different populations of patients affected by HD. ^18^F FDG PET/ceCT significantly improved the diagnostic accuracy and directly affected therapeutic treatment as compared to a pool of patients staged with separate procedures.^17^

As far as spleen and liver illness sites are concerned, our findings are in partial disagreement with those of the previously mentioned studies. We did not find significant differences while comparing ceCT, PET/ldCT, and PET/ceCT results in organs with the exception of 2 patients with spleen lesions. In particular, a patient presented a hypodense lesion with no focal uptake in the ^18^F FDG PET/CT scan whereas another, with no abnormalities in ceCT, showed a focal FDG uptake (Figure 1, Table 1). The detection of a non-FDG avid lesion is not surprising considering that HD lesions of the spleen usually present a focal or diffuse ^18^F FDG uptake.^17,19,20^ In the already cited paper by Picardi et al,^17^ the authors found that diagnostic CT identified at least 1 focal lesion in 17 patients whereas only 7 patients were positive at PET/ldCT. The authors concluded that the detection of subdiaphragmatic lesions by means of PET/ldCT is affected by dimensions and positions.^17^ Especially for the liver and spleen, respiration may affect image evaluation (as in our case study, where the non-FDG avid lesion is located in the upper pole of the spleen as shown in Figure 1).

As compared to the paper of Picardi et al,^17^ our study shows a different number of patients examined (lower in our study) and a different methodology. The relatively small patient cohort would explain why ceCT was not useful in our study.

As for nodal sites, our findings are in partial disagreement with a previous report on a population of patients with HD and NHL.^33^ Despite a good concordance for supradiaphragmatic lymph nodes at staging,^33^ PET/ceCT showed a more accurate nodal status detection for external iliac lymph nodes, internal iliac lymph nodes, and common iliac lymph nodes compared with PET/ldCT.^33^ This is mainly due to the efficacy of ceCT at providing details on lesion locations, morphology, size, and structural changes to adjacent tissues,^34^ especially for small-sized lymph nodes and retroperitoneal lymphatic pathways.^35^ In our study and in the previous cited report of Rodriguez-Vigil et al^20^ (in which only 34% of the entire population was affected by HD), the conjunction of PET/ldCT with ceCT did not improve the diagnostic accuracy at a nodal level. A possible explanation of these discrepancies can be sought in the different lymphoproliferative disorders examined. In the cited study of Morimoto et al,^33^ only 24% of the patients were affected by HD. Aggressive NHL and HD generally show a significantly higher ^18^F FDG uptake than indolent lymphomas^35^; for example, HD and aggressive NHL types have a high uptake of FDG and, given the potentially lower sensitivity for detecting lymphoma deposits, the use of ^18^F FDG-PET for indolent-type lymphomas has been questioned.^35^

Our results show that both PET/ldCT and PET/ceCT are able to detect a larger number of extranodal sites in bone marrow (that is of utmost importance for staging^11^) as compared with ceCT alone. In our study, bone marrow involvement has been described in 8 patients in PET/ldCT and PET/ceCT and only 2 of them presented positive ceCT findings. This last aspect confirms the limitations of ceCT to identify limited skeletal involvement.^36^ Interestingly, in the already cited paper of Pinilla et al,^19^ the authors did not find significant differences while comparing bone marrow sites as detectable by means of ceCT alone with PET/ldCT and PET/ceCT. These results could be explained by the high portion of low-grade histology NHL in the population examined by Pinilla et al^19^; the authors concluded that PET was suboptimal to evaluate the bone marrow in this subgroup of patients.

All the discordant findings in staging HD (3/40 patients, 7.5%) are due to bone marrow sites. It is of interest to note that both PET/ceCT and PET/ldCT mostly upstaged disease when compared with ceCT alone, especially in the early stages of the disease as previously reported.^18^ Further studies are necessary in the more advanced stages of HD in order to confirm the added value of PET at staging.

ceCT allowed the detection of a CCRCC (non-^18^F FDG avid, Figure 3) that could be misdiagnosed in PET/ldCT. In agreement with the results of Pinilla et al,^19^ ceCT could cover a minor role in staging HD due to the incidental findings in PET/ceCT examination.

## CONCLUSIONS

The results of our study suggest that the conjunction of PET/ldCT with ceCT does not impact the staging in patients with HD. PET leads to a higher diagnostic accuracy in staging HD, especially for bone marrow lesions as compared with ceCT alone. The higher radiation exposure because of a ceCT scan could be avoided while staging patients with HD or reserved for selected cases.
